# Synthesis of *μ*-ABC Tricyclic Miktoarm Star Polymer via Intramolecular Click Cyclization

**DOI:** 10.3390/polym10080877

**Published:** 2018-08-06

**Authors:** Tomoki Shingu, Takuya Yamamoto, Kenji Tajima, Takuya Isono, Toshifumi Satoh

**Affiliations:** 1 Graduate School of Chemical Sciences and Engineering, Hokkaido University, Sapporo 060-8628, Hokkaido, Japan; shingu@eis.hokudai.ac.jp; 2 Faculty of Engineering, Hokkaido University, Sapporo 060-8628, Hokkaido, Japan; yamamoto.t@eng.hokudai.ac.jp (T.Y.); ktajima@eng.hokudai.ac.jp (K.T.)

**Keywords:** triblock terpolymer, cyclic polymer, ring-opening polymerization, polyether, click reaction

## Abstract

Cyclic polymers exhibit unique physical and chemical properties because of the restricted chain mobility and absence of chain ends. Although many types of homopolymers and diblock copolymers possessing cyclic architectures have been synthesized to date, there are relatively few reports of cyclic triblock terpolymers because of their synthetic difficulties. In this study, a novel synthetic approach for *μ*-ABC tricyclic miktoarm star polymers involving *t*-Bu-P_4_-catalyzed ring-opening polymerization (ROP) of glycidyl ethers and intramolecular copper-catalyzed azido-alkyne cycloaddition (CuAAC) was developed. First, the *t*-Bu-P_4_-catalyzed ROP of decyl glycidyl ether, dec-9-enyl glycidyl ether, and 2-(2-(2-methoxyethoxy) ethoxy) ethyl glycidyl ether with the aid of functional initiators and terminators was employed for the preparation of a clickable linear triblock terpolymer precursor possessing three azido and three ethynyl groups at the selected positions. Next, the intramolecular CuAAC of the linear precursor successfully produced the well-defined tricyclic triblock terpolymer with narrow dispersity in a reasonable yield. The present strategy is useful for synthesizing model polymers for studying the topological effects on the triblock terpolymer self-assembly.

## 1. Introduction

Block copolymers (BCP) consisting of more than two different polymer segments (or blocks) have attracted considerable attention for their unique self-assembly properties such as microphase-separation and micellization [1,2,3]. It is well known that the molecular weight, volume fraction of each constituting block, and incompatibility between the blocks determine the dimension and morphology of the resulting self-assembled nanostructure. In addition to these classical structural parameters, macromolecular architectures such as star, comb, and cyclic polymer structures, have recently been recognized as an important factor affecting the BCP self-assembly behavior [4]. Several pioneering studies have indicated that cyclic diblock copolymers display unique self-assembly behaviors in both solution and solid states. For example, Tezuka et al. discovered that amphiphilic cyclic poly(ethylene oxide)-*b*-poly(butyl acrylate) formed micellar aggregates with greater thermal stability than the corresponding linear counterpart [5]. Hawker et al. reported that cyclic polystyrene-*b*-poly(ethylene oxide) self-assembled into a hexagonally close-packed cylindrical structure with smaller domain-spacing in the thin film state compared to its corresponding linear counterpart [6]. Thus, further studies relating to cyclic polymer synthesis and self-assembly are highly desired.

Triblock terpolymers consisting of three different polymer segments also self-assemble in both solution and solid states, producing much more complex and diversified nanostructures than those created by diblock copolymers. For example, Noda et al. investigated the morphology of polyisoprene-*b*-polystyrene-*b*-poly(2-vinylpridine) in bulk and discussed the variation in morphologies depending on the composition [7,8]. Moreover, Müller et al. investigated the solution state self-assembly of linear triblock terpolymers to produce various micellar morphologies, such as three-layer core-shell-corona spheres, footballs, and hamburgers [9,10,11] Thus, the combination of a triblock terpolymer system with branched and cyclic architectures is exciting because nanostructures with a variety of novel morphologies and functions are created. Indeed, Dotera et al. simulated the morphology of *μ*-ABC miktoarm star polymers and reported new self-assembled structures that could not have been attained from diblock copolymers or linear triblock terpolymer counterparts [12]. In addition, Matsushita et al. found that *μ*-ABC miktoarm star polymers constructed an Archimedean tiling pattern in the bulk [13]. Ree et al. also found a complex three-phased hexagonal morphology in the asymmetric nine-arm star polymer, (polystyrene)_3_-*b*-(poly(4-methoxystyrene))_3_-*b*-(polyisoprene)_3_ [14]. These self-assembled structures in thin films can be used for lithographic templates for fabricating complex nanopatterns [15,16]. In contrast, triblock terpolymers with cyclic architectures have received scant attention, which is mainly because of their synthetic inaccessibility. In order to study the correlation between the cyclic architecture and self-assembled nanostructures in a triblock terpolymer system, establishing a facile synthetic route toward the architecturally complex triblock terpolymers with well-defined molecular weight and composition is crucial.

**Scheme 1:**
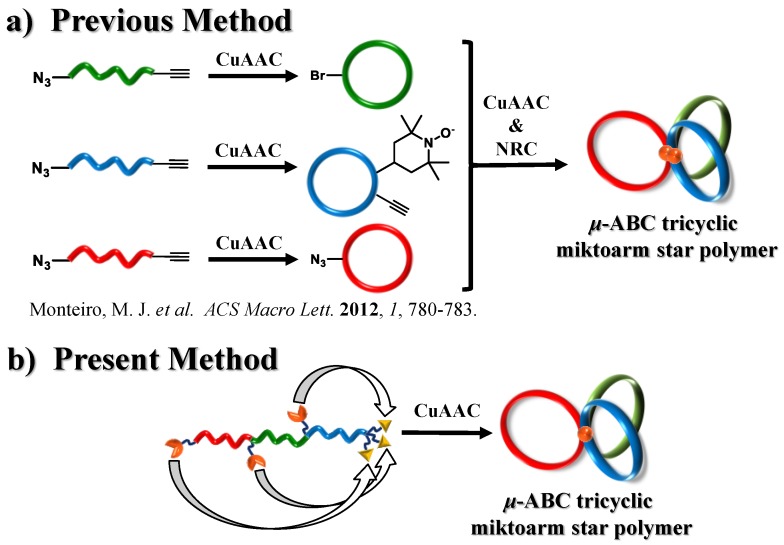
(**a**) Previous and (**b**) present synthetic approaches for *μ*-ABC tricyclic miktoarm star polymers.

Some examples of triblock terpolymers with cyclic architectures are the macrocyclic ABC triblock terpolymer and *μ*-ABC tricyclic miktoarm star polymer. Hadjichristidis et al. successfully synthesized a macrocyclic ABC triblock terpolymer via intramolecular Glaser coupling of a poly(isoprene-*b*-styrene-*b*-2-vinylpyridine) linear precursor [17]. The authors observed a significant influence of the cyclic architecture on the terpolymer microphase separation. Monteiro et al. reported the first synthesis of the *μ*-ABC tricyclic miktoarm star polymer, having macrocyclic polystyrene, poly(*t*-butyl acrylate), and poly(methyl acrylate) units [18]. In this synthesis, three different macrocyclic units possessing a reactive functional group were synthesized by the copper-catalyzed azido-alkyne cycloaddition (CuAAC) [19,20], and then combined via CuAAC and nitroxide radical coupling (NRC) reaction [21] to form the tricyclic structure (Scheme 1a). Although this strategy is highly sophisticated, a challenge still exists in establishing a new strategy for the *μ*-ABC tricyclic miktoarm star polymer without using the intermolecular coupling reaction.

**Scheme 2:**
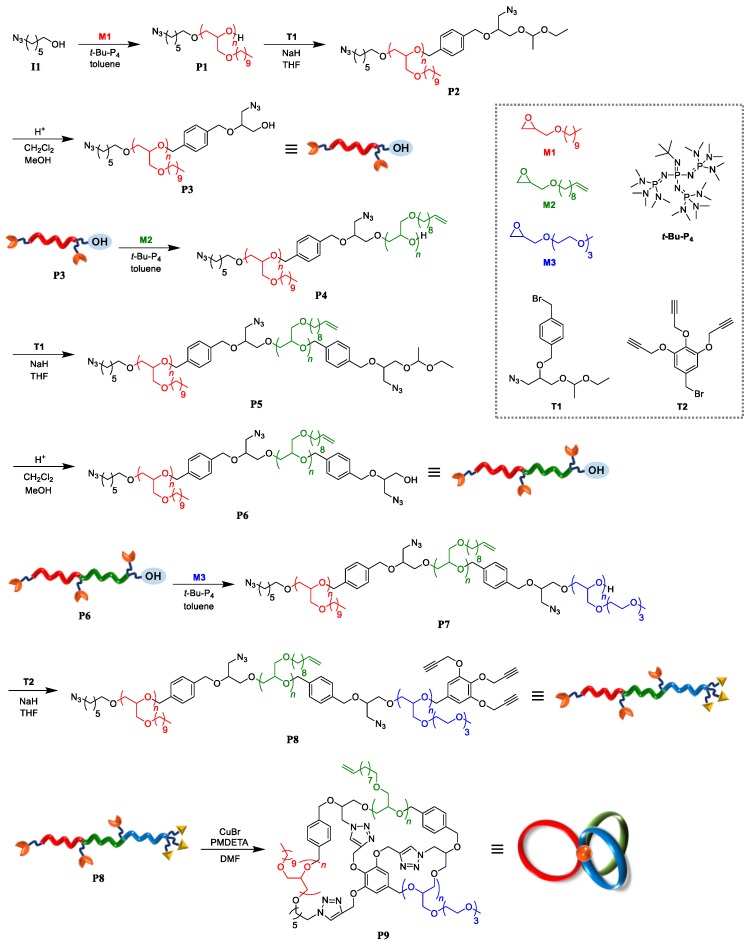
Synthetic pathway for *μ*-ABC tricyclic miktoarm star polymer (**P9**) based on intramolecular click cyclization.

In this study, a novel synthetic approach toward *μ*-ABC tricyclic miktoarm star polymers consisting of a polyether backbone based on intramolecular click cyclization has been proposed (Scheme 1b). The most important feature of the proposed approach is that the three multicyclic units can be constructed in a single click reaction step. In previous studies, figure-eight-, trefoil-, and quatrefoil-shaped block copolymers were synthesized via the intramolecular click reaction [22,23]. Thus, the present strategy should be highly feasible as long as a well-defined clickable precursor can be synthesized. For the synthesis of the clickable precursor, *t*-Bu-P_4_-catalyzed ring-opening polymerization (ROP) of glycidyl ethers was employed as it enabled a precise control over the end group structure and molecular weight. Scheme 2 describes a detailed synthetic pathway for constructing a *μ*-ABC tricyclic miktoarm star polymer (**P9**) consisting of cyclic units of poly(decyl glycidyl ether) (**M1**), poly(dec-9-enyl glycidyl ether) (**M2**), and poly[2-(2-(2-methoxyethoxy) ethoxy) ethyl glycidyl ether] (**M3**). The linear triblock terpolymer possessing three azido groups and three ethynyl groups (**P8**) was synthesized by combining *t*-Bu-P_4_-catalyzed ROP and *ω*-end functionalization. It was then subjected to intramolecular click cyclization to produce **P9**. To the best of our knowledge, this is the first example of the construction of a *μ*-ABC tricyclic miktoarm star polymer via intramolecular coupling.

## 2. Materials and Methods

**Materials.** Decyl glycidyl ether (**M1**) [24], dec-9-enyl glycidyl ether (**M2**) [24], and 2-(2-(2-methoxyethoxy) ethoxy) ethyl glycidyl ether (**M3**) [25] were synthesized according to reported methods, purified by distillation under vacuum over calcium hydride, and stored under an argon atmosphere. 1-(((1-Azido-3-(1-ethoxyethoxy)propan-2-yl)oxy)-methyl)-4-(bromomethyl)benzene (**T1**) [22], 5-(bromomethyl)-1,2,3-tris(prop-2-yn-1-yloxy)benzene (**T2**) [26], 6-azide-1-hexanol (**I1**) [27], and propargyl-functionalized Wang resin (PSt-C≡CH) [28] were synthesized according to reported methods. All other chemicals were used as received. Calcium hydride (CaH_2_) and benzoic acid were purchased from Kanto Chemical Co., Ltd. (Tokyo, Japan), and sodium hydride (NaH, 60%) and *N,N,N*’*,N*’’*,N*’’-pentamethyldiethylenetriamine (PMDETA) were purchased from Tokyo Chemical Industry Co., Ltd. (TCI, Tokyo, Japan). *t*-Bu-P_4_ (1.0 mol·L^−1^ in *n*-hexane), copper (I) bromide (CuBr), aluminum oxide (alumina), and DOWEX^®^ 50WX2 were procured from the Sigma-Aldrich Chemicals Co. (St. Louis, MI, USA). Dry dimethylformamide (DMF, >99.5%; water content, <0.005%), dry toluene (>99.5%; water content, <0.001%), and dry tetrahydrofuran (THF, >99.5%; water content, <0.001%) were purchased from Kanto Chemical Co., Inc.

**Instruments.** The instruments used in this study, such as the glovebox (an MBRAUN stainless steel glovebox, Garching, Germany), size exclusion chromatography (SEC) (a Shodex GPC-101 gel permeation chromatography system, Tokyo, Japan), NMR (a JEOL ECS400 instrument, Tokyo, Japan, 400 MHz), and FT-IR (a PerkinElmer Frontier MIR spectrometer, Waltham, MA, USA) are the same as reported in our previous paper [29].

**Synthesis of azido poly(M1) (P1).** A typical polymerization procedure is as follows (method A): **M1** (1.11 mL, 4.67 mmol) was added to a stirred solution of **I1** (141 µL, 141 µmol) and *t*-Bu-P_4_ (141 µL of 1.0 mol·L^−1^ solution in *n*-hexane, 141 µmol) in toluene (478 µL), and then the entire mixture was stirred at r.t. (room temperature) for 20 h. Subsequently, an excess of benzoic acid was added to the mixture to terminate the polymerization, and the product was purified by passing it through a pad of alumina using THF to obtain **P1** as a colorless viscous liquid (558 mg). Yield: 56.5%. ^1^H NMR (400 MHz, CDCl_3_): *δ* (ppm) 3.29–3.75 (m, N_3_(CH_2_)_5_C*H*_2_OCH_2_–, N_3_(CH_2_)_6_OCH_2_–, –OCH_2_CH(CH_2_OCH_2_(CH_2_)_8_CH_3_)O–), 3.85–3.95 (s, –OCH_2_CH(CH_2_OCH_2_(CH_2_)_8_CH_3_)OH), 3.26 (t, N_3_CH_2_–), 1.10–1.69 (m, N_3_CH_2_(CH_2_)_4_CH_2_O–, –OCH_2_CH(CH_2_OCH_2_CH_2_(CH_2_)_7_CH_3_)O–), and 0.78–0.95 (t, –OCH_2_CH(CH_2_OCH_2_(CH_2_)_8_CH_3_)O–). *M*_n,NMR_ = 7000 g·mol^−1^ (CDCl_3_); *M*_n,SEC_ = 7400 g·mol^−1^ (THF); *Đ* = 1.03.

**Synthesis of diazido poly(M1) (P2).** A typical procedure for the *ω*-chain end functionalization is as follows (method B): Under nitrogen atmosphere, NaH (47.8 mg, 1.19 mmol) was added to a stirred solution of **P1** (*M*_n,NMR_ = 7000 g·mol^−1^, 558 mg) in THF (5.0 mL) and the entire mixture was stirred at 40 °C for 30 min. **T1** (296 mg, 796 µmol) was then added to the reaction mixture, which was stirred at r.t. for 48 h under nitrogen. The product was purified by passing it through a pad of alumina to obtain **P2** as a pale yellow viscous liquid (489 mg). Yield: 75.4%. ^1^H NMR (400 MHz, CDCl_3_): *δ* (ppm) 7.36 (aromatic), 4.70 (m, –CH_2_PhCH_2_O–), 4.64 (s, –OCH(CH_3_)O–), 3.29–3.75 (m, N_3_(CH_2_)_5_C*H*_2_OCH_2_−, –OCH_2_CH(CH_2_OCH_2_(CH_2_)_8_CH_3_)O–, N_3_CH_2_CH(O–)CH_2_–, –C*H*_2_OCH(CH_3_)OCH_2_CH_3_), 3.26 (t, N_3_CH_2_CH_2_–), 1.10–1.69 (m, N_3_CH_2_(CH_2_)_4_CH_2_O–, –OCH_2_CH(CH_2_OCH_2_CH_2_(CH_2_)_7_CH_3_)O–), and 0.78–0.95 (t, –OCH_2_CH(CH_2_OCH_2_(CH_2_)_8_CH_3_)O–). *M*_n,NMR_ = 7950 g mol^−^^1^ (CDCl_3_); *M*_n,SEC_ = 8000 g mol^−^^1^ (THF); *Đ* = 1.03.

**Synthesis of diazido-hydroxyl poly(M1) (P3).** A typical procedure for the deprotection of ethoxyethyl group is as follows (method C): DOWEX^®^ (100 mg) and distilled water (100 µL) were added to the stirred solution of **P2** (*M*_n,NMR_ = 7950 g·mol^−1^, 489 mg) in CH_2_Cl_2_/MeOH (10 mL, *v*/*v* = 4/1), and the entire mixture was stirred at r.t. for 48 h. The product was purified by passing it through a pad of alumina to obtain **P3** as a pale yellow viscous liquid (331 mg). Yield: 70.6%. ^1^H NMR (400 MHz, CDCl_3_): *δ* (ppm) 7.36 (aromatic), 4.70 (m, –CH_2_PhCH_2_O–), 3.29–3.75 (m, N_3_(CH_2_)_5_CH_2_OCH_2_–, –OCH_2_CH(CH_2_OCH_2_(CH_2_)_8_CH_3_)O–, N_3_CH_2_CH(O–)CH_2_–, –CH_2_OH), 3.26 (t, N_3_CH_2_–), 1.10–1.69 (m, N_3_CH_2_(CH_2_)_4_CH_2_O–, –OCH_2_CH(CH_2_OCH_2_CH_2_(CH_2_)_7_CH_3_)O–), and 0.78–0.95 (t, –OCH_2_CH(CH_2_OCH_2_(CH_2_)_8_CH_3_)O–). *M*_n,NMR_ = 7810 g·mol^−1^ (CDCl_3_); *M*_n,SEC_ = 7500 g·mol^−1^ (THF); *Đ* = 1.03.

**Synthesis of poly(M1)-*b*-poly(M2) (P4).** Method A was used for the polymerization of **M2** (273 µL, 35.6 µmol) with **P3** (*M*_n,NMR_ = 7810 g·mol^−1^, 331 mg) and *t*-Bu-P_4_ (35.6 µL of 1.0 mol·L^−1^ solution in *n*-hexane, 35.6 µmol) in toluene (478 µL) to obtain **P4** as a pale yellow viscous liquid (533 mg). Yield: 84.7%. ^1^H NMR (400 MHz, CDCl_3_): *δ* (ppm) 7.36 (aromatic), 5.71–5.90 (q, –OCH_2_CH(CH_2_OCH_2_(CH_2_)_6_CH=CH_2_)O–), 4.86–5.07 (d, –OCH_2_CH(CH_2_OCH_2_(CH_2_)_6_CH=CH_2_)O–), 4.56–4.77 (m, –CH_2_PhCH_2_O–), 3.29–3.85 (m, N_3_(CH_2_)_5_C*H*_2_OCH_2_–, –OCH_2_CH(CH_2_OCH_2_(CH_2_)_8_CH_3_)O–, –OCH_2_CH(CH_2_OCH_2_(CH_2_)_6_CH=CH_2_)O–, N_3_CH_2_CH(O–)CH_2_–), 3.21–3.28 (t, N_3_CH_2_–), 1.14–1.80 (m, N_3_CH_2_(CH_2_)_4_CH_2_O–, –OCH_2_CH(CH_2_OCH_2_CH_2_(CH_2_)_7_CH_3_)O–, –OCH_2_CH(CH_2_OCH_2_(CH_2_)_6_CH=CH_2_)O–), and 0.78–0.98 (t, –OCH_2_CH(CH_2_OCH_2_(CH_2_)_8_CH_3_)O–). *M*_n,NMR_ = 14,600 g·mol^−1^ (CDCl_3_); *M*_n,SEC_ = 12,500 g·mol^−1^ (THF); *Đ* = 1.10.

**Synthesis of triazido poly(M1)-*b*-poly(M2) (P5).** Method B was used for the *ω*-chain end functionalization of **P4** (*M*_n,NMR_ = 14,600 g·mol^−1^, 533 mg) with NaH (21.2 mg, 530 µmol) and **T1** (131 mg, 353 µmol) in THF (5.0 mL) to obtain **P5** as a pale yellow viscous liquid (485 mg). Yield: 90.8%. ^1^H NMR (400 MHz, CDCl_3_): *δ* (ppm) 7.36 (aromatic), 5.72–5.85 (q, –OCH_2_CH(CH_2_OCH_2_(CH_2_)_6_CH=CH_2_)O–), 4.87–5.02 (d, –OCH_2_CH(CH_2_OCH_2_(CH_2_)_6_CH=CH_2_)O–), 4.55–4.77 (m, –CH_2_PhCH_2_O–), 3.29–3.74 (m, N_3_(CH_2_)_5_CH_2_OCH_2_–, –OCH_2_CH(CH_2_OCH_2_(CH_2_)_8_CH_3_)O–, –OCH_2_CH(CH_2_OCH_2_(CH_2_)_6_CH=CH_2_)O–, N_3_CH_2_CH(O–)CH_2_–), 3.21–3.28 (t, N_3_CH_2_–), 1.14–1.75 (m, N_3_CH_2_(CH_2_)_4_CH_2_O–, –OCH_2_CH(CH_2_OCH_2_CH_2_(CH_2_)_7_CH_3_)O–, –OCH_2_CH(CH_2_OCH_2_(CH_2_)_6_CH=CH_2_)O–), and 0.78–0.96 (t, –OCH_2_CH(CH_2_OCH_2_(CH_2_)_8_CH_3_)O–). *M*_n,NMR_ = 14,800 g·mol^−1^ (CDCl_3_); *M*_n,SEC_ = 12,700 g·mol^−1^ (THF); *Đ* = 1.08.

**Synthesis of triazido-hydroxyl poly(M1)-*b*-poly(M2) (P6).** Method C was used for the deprotection of **P5** (*M*_n,NMR_ = 14,800 g·mol^−1^, 485 mg) with DOWEX^®^ (100 mg) and distilled water (100 µL) in CH_2_Cl_2_/MeOH (10 mL, *v*/*v* = 4/1). The residue was purified by preparative SEC to obtain **P6** as a pale yellow viscous liquid (168 mg). Yield: 34.5%. ^1^H NMR (400 MHz, CDCl_3_): *δ* (ppm) 7.36 (aromatic), 5.72–5.85 (q, –OCH_2_CH(CH_2_OCH_2_(CH_2_)_6_CH=CH_2_)O–), 4.87–5.02 (d, –OCH_2_CH(CH_2_OCH_2_(CH_2_)_6_CH=CH_2_)O–), 4.55–4.77 (m, –CH_2_PhCH_2_O–), 3.29–3.74 (m, N_3_(CH_2_)_5_CH_2_OCH_2_–, –OCH_2_CH(CH_2_OCH_2_(CH_2_)_8_CH_3_)O–, –OCH_2_CH(CH_2_OCH_2_(CH_2_)_6_CH=CH_2_)O–, N_3_CH_2_CH(O–)CH_2_–, –CH_2_OH), 3.21–3.28 (t, N_3_CH_2_–), 1.14–1.75 (m, N_3_CH_2_(CH_2_)_4_CH_2_O–, –OCH_2_CH(CH_2_OCH_2_CH_2_(CH_2_)_7_CH_3_)O–, –OCH_2_CH(CH_2_OCH_2_(CH_2_)_6_CH=CH_2_)O–), and 0.78–0.96 (t, –OCH_2_CH(CH_2_OCH_2_(CH_2_)_8_CH_3_)O–). *M*_n,NMR_ = 14,400 g·mol^−1^ (CDCl_3_); *M*_n,SEC_ = 12,600 g·mol^−1^ (THF); *Đ* = 1.04.

**Synthesis of poly(M1)-*b*-poly(M2)-*b*-poly(M3) (P7).** Method A was used for polymerization of **M3** (62 µL, 299 µmol) with **P6** (*M*_n,NMR_ = 14,400 g·mol^−1^, 131 mg) and *t*-Bu-P_4_ (9.1 µL of 1.0 mol·L^−1^ solution in *n*-hexane, 9.1 µmol) in toluene (98 µL). The residue was purified by preparative SEC to obtain **P7** as a pale yellow viscous liquid (127 mg). Yield: 80.4%. ^1^H NMR (400 MHz, CDCl_3_): *δ* (ppm) 7.36 (aromatic), 5.72–5.85 (q, –OCH_2_CH(CH_2_OCH_2_(CH_2_)_6_CH=CH_2_)O–), 4.87–5.02 (d, –OCH_2_CH(CH_2_OCH_2_(CH_2_)_6_CH=CH_2_)O–), 4.55–4.72 (m, –CH_2_PhCH_2_O–), 3.29–3.74 (m, N_3_(CH_2_)_5_CH_2_OCH_2_–, –OCH_2_CH(CH_2_OCH_2_(CH_2_)_8_CH_3_)O–, –OCH_2_CH(CH_2_OCH_2_(CH_2_)_6_CH=CH_2_)O–, –OCH_2_CH(CH_2_O(CH_2_CH_2_O)_3_CH_3_)O–, N_3_CH_2_CH(O–)CH_2_–), 3.21–3.28 (t, N_3_CH_2_–), 1.15–1.75 (m, N_3_CH_2_(CH_2_)_4_CH_2_O–, –OCH_2_CH(CH_2_OCH_2_CH_2_(CH_2_)_7_CH_3_)O–, –OCH_2_CH(CH_2_OCH_2_(CH_2_)_6_CH=CH_2_)O–), and 0.75–0.93 (t, –OCH_2_CH(CH_2_OCH_2_(CH_2_)_8_CH_3_)O–). *M*_n,NMR_ = 19,600 g·mol^−1^ (CDCl_3_); *M*_n,SEC_ = 14,200 g·mol^−1^ (THF); *Đ* = 1.03.

**Synthesis of triazido-triethynylpoly(M1)-*b*-poly(M2)-*b*-poly(M3) (P8).** Method B was used for the *ω*-chain end functionalization of **P7** (*M*_n, NMR_ = 19,600 g·mol^−1^, 50 mg) with NaH (1.51 mg, 37.8 µmol) and **T2** (8.39 mg, 25.2 µmol) in THF (5.0 mL). The residue was purified by preparative SEC to obtain **P8** as a pale yellow viscous liquid (43 mg). Yield: 90.8%. ^1^H NMR (400 MHz, CDCl_3_): *δ* (ppm) 7.36 (aromatic), 5.72–5.85 (q, –OCH_2_CH(CH_2_OCH_2_(CH_2_)_6_CH=CH_2_)O–), 4.87–5.02 (d, –OCH_2_CH(CH_2_OCH_2_(CH_2_)_6_CH=CH_2_)O–), 4.55–4.77 (m, –CH_2_PhCH_2_O–, –CH_2_OPhOCH_2_C≡CH), 3.28–3.75 (m, N_3_(CH_2_)_5_CH_2_OCH_2_–, –OCH_2_CH(CH_2_OCH_2_(CH_2_)_8_CH_3_)O–,–OCH_2_CH(CH_2_OCH_2_(CH_2_)_6_CH=CH_2_)O–, –OCH_2_CH(CH_2_O(CH_2_CH_2_O)_3_CH_3_)O–, N_3_CH_2_CH(O–)CH_2_–), 3.21–3.28 (t, N_3_CH_2_–), 2.57–2.64 (s, m–OCH_2_C≡CH), 2.44–2.49 (s, p–OCH_2_C≡CH), 1.15–1.75 (m, N_3_CH_2_(CH_2_)_4_CH_2_O–, –OCH_2_CH(CH_2_OCH_2_CH_2_(CH_2_)_7_CH_3_)O–, –OCH_2_CH(CH_2_OCH_2_(CH_2_)_6_CH=CH_2_)O–), and 0.75–0.93 (t, –OCH_2_CH(CH_2_OCH_2_(CH_2_)_8_CH_3_)O–). *M*_n,NMR_ = 19,900 g·mol^−1^ (CDCl_3_); *M*_n,SEC_ = 14,700 g·mol^−1^ (THF); *Đ* = 1.04.

**Synthesis of*μ*-ABC tricyclic miktoarm star polymer (P9).** A solution of **P8** (*M*_n,NMR_ = 19,900 g·mol^−1^, 43 mg) in degassed DMF (2.25 mL) was added to a stirred solution of CuBr (30.8 mg, 215 µmol) and PMDETA (89.8 µL, 430 µmol) in degassed DMF (100 mL) using a syringe pump at the rate of 0.3 mL·h^−1^ at 100 °C under a nitrogen atmosphere. Next, PSt-C≡CH (10 mg), CuBr (6.2 mg, 43 µmol), and PMDETA (18.0 µL, 86 µmol) were added to the reaction mixture. After stirring at 100 °C for 24 h, the solvent was removed by evaporation, and the residue was purified by passing it through a pad of alumina. This was followed by preparative SEC to obtain **P9** as a pale yellow viscous liquid (22 mg). Yield: 50.7%. ^1^H NMR (400 MHz, CDCl_3_): *δ* (ppm) 7.64–7.94 (triazole methine), 7.19–7.32, 6.62–6.86 (aromatic), 5.72–5.85 (q, –OCH_2_CH(CH_2_OCH_2_(CH_2_)_6_CH=CH_2_)O–), 5.05–5.26 (m, –triazole ring–CH_2_OPh–), 4.87–5.02 (d, –OCH_2_CH(CH_2_OCH_2_(CH_2_)_6_CH=CH_2_)O–), 4.37–4.73 (m, –CH_2_PhCH_2_O–, –CH_2_OPhOCH_2_–triazole ring–, –OCH(CH_2_–triazole ring–)CH_2_O–), 4.22–4.34 (m, –triazole ring–CH_2_(CH_2_)_4_CH_2_O–), 3.88–4.03 (m, –OCH(CH_2_–triazole ring–)CH_2_O–), 3.28–3.75 (m, –triazole ring–CH_2_(CH_2_)_4_CH_2_O–, –OCH_2_CH(CH_2_OCH_2_(CH_2_)_8_CH_3_)O–, –OCH_2_CH(CH_2_OCH_2_(CH_2_)_6_CH=CH_2_)O–, –OCH_2_CH(CH_2_O(CH_2_CH_2_O)_3_CH_3_)O–, –OCH(CH_2_–triazole ring–)CH_2_O–), 1.15–1.75 (m, –triazole ring–CH_2_(CH_2_)_4_CH_2_O–, –OCH_2_CH(CH_2_OCH_2_CH_2_(CH_2_)_7_CH_3_)O–, –OCH_2_CH(CH_2_OCH_2_(CH_2_)_6_CH=CH_2_)O–), and 0.75–0.93 (t, –OCH_2_CH(CH_2_OCH_2_(CH_2_)_8_CH_3_)O–). *M*_n,NMR_ = 20,100 g·mol^−1^ (CDCl_3_); *M*_n,SEC_ = 11,500 g·mol^−1^ (THF); *Đ* = 1.02.

## 3. Results and Discussion

**Figure 1:**
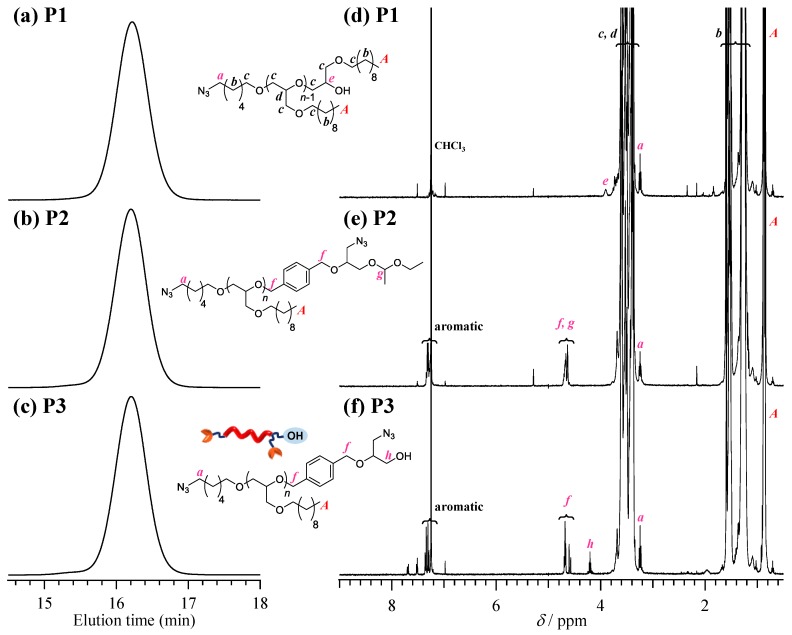
(**a**–**c**) Size exclusion chromatography (SEC) traces of **P1**, **P2**, and **P3**. (**d**–**f**) ^1^H NMR spectra of **P1**, **P2**, and **P3** in CDCl_3_ (400 MHz).

**Figure 2:**
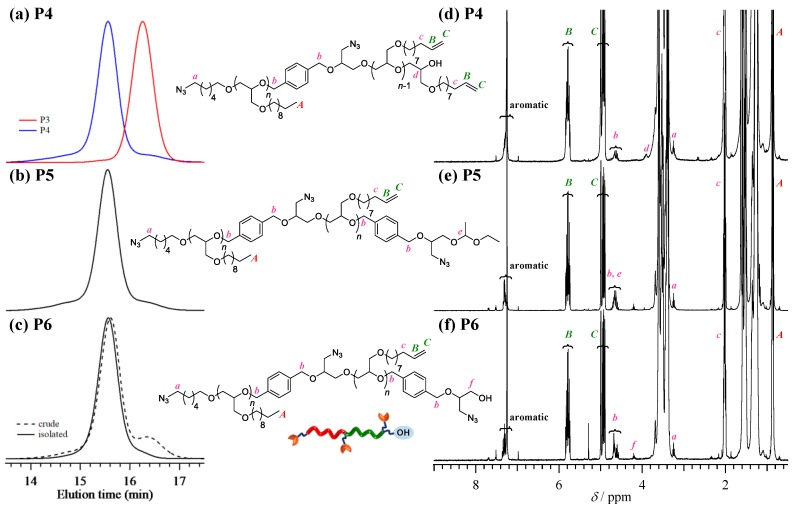
(**a**–**c**) SEC traces of **P4** (red line, first polymerization of **M1**; blue line, after the block copolymerization of **M2**), **P5**, and **P6** (dotted line, before purification; solid line, after purification using preparative SEC). (**d**–**f**) ^1^H NMR spectra of **P1**, **P2**, and **P3** in CDCl_3_ (400 MHz).

**Synthesis of diazido-hydroxyl poly(M1) (P3)**. As the first step of the synthetic route, diazido-hydroxyl poly(**M1**) (**P3**) was synthesized in three steps, namely, the polymerization of decyl glycidyl ether (**M1**) with 6-azido-1-hexanol (**I1**), end group modification with 1-(((1-azido-3-(1-ethoxyethoxy)propan-2-yl)oxy)-methyl)-4-(bromomethyl)benzene (**T1**), and deprotection of the ethoxyethyl group (Scheme 2). Following a previous report [22], the *t*-Bu-P_4_-catalyzed ROP of **M1** using **I1** as an initiator was carried out at the [**M1**]_0_/[**I1**]_0_/[*t*-Bu-P_4_] ratio of 33/1/1 to produce azido poly(**M1**) (**P1**; *M*_n,NMR_ = 7000 g·mol^−1^, degree of polymerization for block: DP_1_ = 33, molecular weight dispersion: *Đ* = 1.03) in 56.5% isolated yield. The ^1^H NMR spectrum of **P1** showed the characteristic signals corresponding to the poly(**M1**) backbone along with minor signals of the initiator residue, such as the methylene groups adjacent to the azido groups (*A*: 3.26 ppm in Figure 1d), verifying that the ROP of **M1** was initiated from **I1**. The number-average molecular weight determined from NMR analysis (*M*_n,NMR_) of **P1** was in good agreement with the *M*_n_ value (*M*_n,theo_) calculated by the monomer conversion and the initial monomer-to-initiator ratio (*M*_n,theo_ = 7220) (Table 1). Next, **P1** was treated with an excess amount of **T1** in the presence of sodium hydride to obtain diazido poly(**M1**) (**P2**; *M*_n,NMR_ = 7,950 g·mol^−1^, DP_1_ = 33, *Đ* = 1.03). After *ω*-end functionalization, the ethoxyethyl group of **P2** was deprotected under acidic conditions to give diazido-hydroxyl poly(**M1**) (**P3**; *M*_n,NMR_ = 7810 g·mol^−1^, DP = 33, *Đ* = 1.03). After thorough screening of the deprotection conditions, it was found that the cation exchange resin (DOWEX^®^ hydrogen form) was the best suited for a clean reaction without undesired side reactions. There was no significant difference between the SEC traces of the **P1**, **P2**, and **P3**, suggesting the absence of any side reactions (Figure 2a–c). The ^1^H NMR spectrum of **P3** showed the signals of the benzyl protons (protons *f* in Figure 2e,f) at 4.70 ppm as well as methylene protons adjacent to the azido group (protons *a*) at 3.26 ppm, based on which the introduction of an azido and a hydroxyl group at the *ω*-chain end was verified.

**Synthesis of triazido-hydroxyl poly(M1)-*b*-poly(M2) (P6).** After rigorous dehydration, **P3** was utilized as a macroinitiator for the synthesis of poly(**M1**)-*b*-poly(**M2**) (**P4**; *M*_n,NMR_ = 14,600 g·mol^−1^, DP_1_/DP_2_ = 33/33, *Đ* = 1.10). The *t*-Bu-P_4_-catalyzed ROP of dec-9-enyl glycidyl ether (**M2**) with **P3** macroinitiator was carried out at the [**M2**]_0_/[**P3**]_0_/[*t*-Bu-P_4_] ratio of 33/1/1 to obtain **P4** in 84.7% yield. The SEC trace of **P3** shifted to the higher molecular weight region after polymerization (Figure 2a), which confirmed that the polymerization reaction was initiated from the hydroxyl group of **P3**. The ^1^H NMR spectrum of **P3** showed characteristic signals of both the poly(**M2**) and poly(**M1**) backbones, verifying successful post polymerization (Figure 2d). In a similar fashion to the synthesis of **P3**, **P4** was treated with **T1** in the presence of sodium hydride, and the ethoxyethyl group was deprotected under acidic conditions to give triazido-hydroxyl poly(**M1**)-*b*-poly(**M2**) (**P6**). It should be noted that a non-negligible amount of a lower molecular weight byproduct was observed in the SEC trace of crude **P6** (Figure 2c). It was expected that the residue of macroinitiator **P3**, which was not completely deprotected, would correspond to this shoulder peak. According to the SEC profile, 5.0% of the macroinitiator did not participate in the polymerization reaction (Figure 2a). To remove the unreacted macroinitiator, the crude product was subjected to preparative SEC and pure **P6** was isolated in 34.5% yield (*M*_n,NMR_ = 14,400 g·mol^−1^, DP_1_/DP_2_ = 33/33, *Đ* = 1.04) (Table 2).

**Figure 3:**
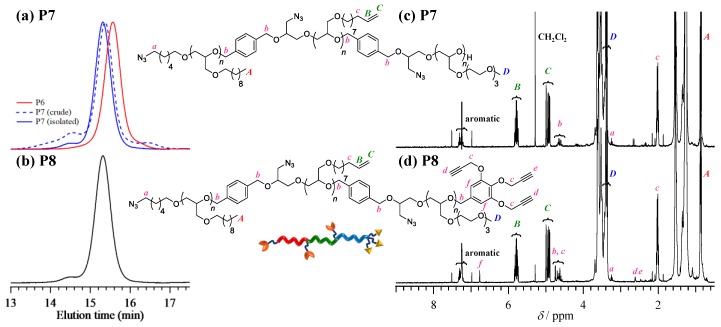
(**a**,**b**) SEC traces of **P7** (dotted line, before purification; solid line, after purification using preparative SEC) and **P8**. (**c**,**d**) ^1^H NMR spectra of **P7** and **P8** in CDCl_3_ (400 MHz).

**Synthesis of triazido-triethynylpoly(M1)-*b*-poly(M2)-*b*-poly(M3) (P8).** In a similar fashion to the synthesis of **P4**, the *t*-Bu-P_4_-catalyzed ROP of 2-(2-(2-methoxyethoxy) ethoxy) ethyl glycidyl ether (**M3**) was carried out at the [**M3**]_0_/[**P6**]_0_/[*t*-Bu-P_4_] ratio of 33/1/1 using **P6** as a macroinitiator for the synthesis of poly(**M1**)-*b*-poly(**M2**)-*b*-poly(**M3**) (**P7**). The success of the extension of a poly(**M3**) block from the macroinitiator **P6** was confirmed by the fact that the elution peak maximum of **P6** shifted to the higher molecular weight region (Figure 3a). However, the SEC trace of the obtained crude product **P7** showed a non-negligible amount of higher and lower molecular weight byproducts, along with the main product. The population of the higher and lower molecular weight byproducts was calculated to be 4.4% and 15.2%, respectively, based on the SEC elution peak area. The low molecular weight byproduct corresponded to the poly(**M3**) homopolymer that was produced by the ROP of **M3** initiated from water contaminant in the monomer or macroinitiator. On the other hand, the high molecular weight byproduct was possibly one of the intermolecularly cross-linked products formed through the reaction between the growing oxyanion and side chain olefin [30]. Thus, the crude product was purified by preparative SEC and pure **P7** was isolated in 80.4% yield (*M*_n,NMR_ = 19,600 g·mol^−1^, DP_1_/DP_2_/DP_3_ = 33/33/25, *Đ* = 1.03) (Table 3). After purification, **P7** was treated with an excess amount of 5-(bromomethyl)-1,2,3-tris(prop-2-yn-1-yloxy)benzene (**T2**) in the presence of sodium hydride to obtain triazido-triethynyl poly(**M1**)-*b*-poly(**M2**)-*b*-poly(**M3**) (**P8**; *M*_n,NMR_ = 19,900 g·mol^−1^, DP_1_/DP_2_/DP_3_ = 33/33/25, *Đ* = 1.04). The proton signal corresponding to the terminal alkynes (protons *d* and *e*) was observed at 2.44–2.64 ppm in the ^1^H NMR spectrum (Figure 3d), and the quantitative introduction of the propargyl groups was verified by comparing the peak areas of the ethynyl protons *d* and *e* (2.44–2.64 ppm) with the benzyl proton *b* (4.45 ppm).

**Figure 4:**
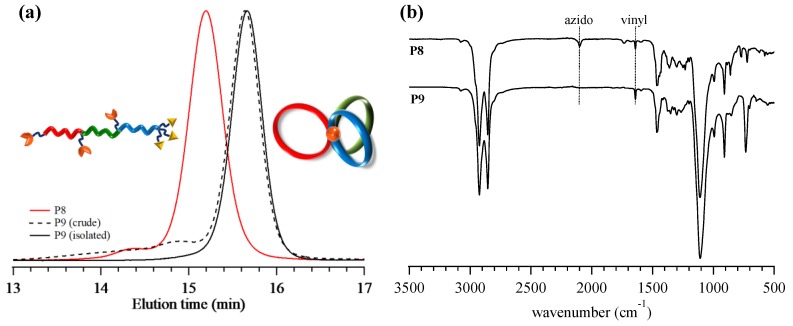
(**a**) SEC traces of **P8** (red line) and **P9** (dotted line, before purification; solid line, after purification using preparative SEC). (**b**) FT-IR spectra of **P8** (upper) and **P9** (lower).

**Figure 5:**
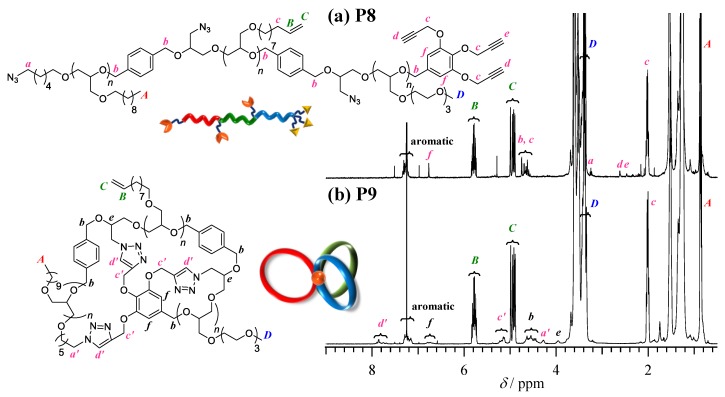
(**a**,**b**) ^1^H NMR spectra of **P8** and **P9** in CDCl_3_ (400 MHz).

**Synthesis of*μ*-ABC tricyclic miktoarm star polymer (P9).** Finally, the intramolecular multiple click cyclization of **P8** was performed to obtain the target *μ*-ABC tricyclic miktoarm star polymer (**P9**) using the CuBr/*N*,*N*,*N*′,*N*″,*N*″-pentamethyldiethylenetriamine (PMDETA) catalyst system in DMF at 100 °C. To avoid the intermolecular click reaction, the slow addition technique was employed. Thus, the **P8** solution in DMF (19.1 mg·mL^−1^) was added slowly to the catalyst solution using the syringe pump at the rate of 0.3 mL·h^−1^. After complete addition, the reaction was continued for another 24 h at 100 °C. Finally, an alkyne-functionalized Wang resin was added to the reaction mixture, by which the unreacted **P8** and any other possible byproducts possessing azido groups were removed by the click reaction. FT-IR analysis of the crude product obtained after the alumina column revealed the complete disappearance of the azido groups (Figure 4b). Notably, the absorption band corresponding to the side chain vinyl groups remained after the click reaction, which indicated that there was no significant side reaction. The crude product was then subjected to SEC analysis to confirm the progress of the cyclization reaction (Figure 4a). The elution peak maximum of the product was observed in the lower molecular weight region as compared to the linear precursor **P8**, which strongly supported the expected decrease in the hydrodynamic volume by the intramolecular cyclization reaction. On the other hand, small broad peaks were visible in the higher molecular weight region, which could be attributed to the oligomeric byproducts formed by the intermolecular click reaction. The population of the intramolecularly cyclized product was calculated to be 85.7% based on the SEC elution peak area. Further purification was then performed by the preparative SEC to remove the high molecular weight byproducts, giving pure product in 53.3% yield. The isolated product displayed a unimodal SEC trace with the *Đ* value of 1.02 (Figure 4a). The ratio between the *M*_n,SEC_s at the SEC peak top of **P9** and **P8**, that is, *M*_n,p(P9)_/*M*_n,p(P8)_ = <*G*>, was calculated to be 0.79 (Table 4). In the ^1^H NMR spectrum, new signals (*a*′, 4.22–4.34 ppm; *c*′, 5.05−5.26 ppm; *d*′, 7.64–7.94 ppm in Figure 5a,b) assignable to the triazole rings formed by the click reaction appeared, while the signals corresponding to the ethynyl and methylene groups adjacent to the azido groups completely disappeared. As results of SEC, FT-IR, and ^1^H NMR analyses all indicated a successful intramolecular click reaction, the product was identified as **P9**. On the basis of end group analysis by ^1^H NMR, the *M*_n,NMR_ and DP_1_/DP_2_/DP_3_ were calculated to be 20,100 g·mol^−1^ and 33/33/25, respectively.

## 4. Conclusions

A new synthetic strategy for the *μ*-ABC tricyclic miktoarm star polymer comprising three different cyclic units of polyethers, namely, poly(decyl glycidyl ether), poly(dec-9-enyl glycidyl ether), and poly[2-(2-(2-methoxyethoxy) ethoxy) ethyl glycidyl ether], has been developed. The *t*-Bu-P_4_-catalyzed ROP of glycidyl ethers was employed for the preparation of a clickable linear triblock terpolymer precursor possessing three azido and three ethynyl groups at the selected positions. The intramolecular multiple click cyclization of the linear precursors successfully produced the well-defined tricyclic triblock terpolymer with narrow dispersity in a reasonable yield. Given the functional group loading capacity of the poly(glycidyl ether), the present strategy can provide model polymers suitable for studying the topological effects on the triblock terpolymer self-assembly. Indeed, the poly(dec-9-enyl glycidyl ether) segment has a reactive olefinic side chain [31] that can be transformed into a variety of functionalities via thiol-ene reaction, epoxidation, hydroboration, and hydrosilylation. Such side chain modification would permit the present terpolymer to self-assemble into three-phase microphase-separated structures, which can be used as templates for constructing complex nanopatterns. Efforts toward the synthesis of a series of triblock terpolymers with various architectures, including linear, star, and cyclic structures, are currently underway in order to comprehensively understand the correlation between the macromolecular architecture and microphase separation in triblock terpolymer systems.

## Figures and Tables

**Table 1:** Characteristics of P1, P2, and P3.

Polymer	*M*_n,NMR_ (g·mol^−1^) *^c^*	*M*_n,SEC_ (g·mol^−1^) *^d^*	*Đ ^d^*	DP_1_ *^b^*	Yield (%)
P1 *^a^*	7000	7400	1.03	33	56.5
P2	7950	8000	1.03	33	75.4
P3	7810	7500	1.03	33	70.6

*^a^* Polymerization conditions: Ar atmosphere; [**M1**]_0_ = 2.5 mol·L^−1^; [**M1**]_0_/[**I1**]_0_/[*t*-Bu-P_4_] = 33/1/1; solvent, toluene; initiator, 6-azide-1-hexanol (**I1**); temp., r.t.; time, 20 h. *^b^* Calculated based on the monomer conversion. *^c^* Determined by ^1^H NMR in CDCl_3_. *^d^* Determined by SEC in THF using polystyrene standards.

**Table 2:** Characteristics of **P4**, **P5**, and **P6**.

Polymer	*M*_n,NMR_ (g·mol^−1^) *^c^*	*M*_n,SEC_ (g·mol^−1^) *^d^*	*Đ ^d^*	DP_1_/DP_2_ *^b^*	Yield (%)
P4 *^a^*	14,600	12,500	1.10	33/33	84.7
P5	14,800	12,700	1.08	33/33	90.8
P6	14,400	12,600	1.04	33/33	34.5

*^a^* Polymerization conditions: Ar atmosphere; [**M2**]_0_ = 1.25 mol·L^−1^; [**M2**]_0_/[**P3**]_0_/[*t*-Bu-P_4_] = 33/1/1; solvent, toluene; temp., r.t.; time, 20 h. *^b^* Calculated based on the monomer conversion. *^c^* Determined by ^1^H NMR in CDCl_3_. *^d^* Determined by SEC in THF using polystyrene standards.

**Table 3:** Characteristics of **P7** and **P8**.

Polymer	*M*_n,NMR_ (g·mol^−1^) *^c^*	*M*_n,SEC_ (g·mol^−1^) *^d^*	*Đ ^d^*	DP_1_/DP_2_/DP_3_ *^b^*	Yield (%)
P7 *^a^*	19,600	14,200	1.03	33/33/25	80.4
P8	19,900	14,700	1.04	33/33/25	90.8

*^a^* Polymerization condition: Ar atmosphere; [**M3**]_0_ = 1.00 mol·L^−1^; [**M3**]_0_/[**P6**]_0_/[*t*-Bu-P_4_] = 25/1/1; solvent, toluene; temp., r.t.; time, 20 h. *^b^* Calculated based on the monomer conversion. *^c^* Determined by ^1^H NMR in CDCl_3_. *^d^* Determined by SEC in THF using polystyrene standards.

**Table 4:** Characteristics of **P8** and **P9**.

Polymer	*M*_n,NMR_ (g·mol^−1^) *^b^*	*M*_n,SEC_ (g·mol^−1^) *^c^*	*Đ ^c^*	DP_1_/DP_2_/DP_3_ *^a^*	<*G*> *^c^*	Yield (%)
P8	19,900	14,700	1.04	33/33/25	0.79	–
P9	20,100	11,500	1.02	33/33/25		53.3

*^a^* Calculated based on the monomer conversion. *^b^* Determined by ^1^H NMR in CDCl_3_. *^c^* Determined by SEC in THF using polystyrene standards.
